# Pharmacokinetic Study of Coadministration with Cefuroxime Sodium for Injection Influencing ReDuNing Injection-Derived Seven Phytochemicals and Nine Metabolites in Rats

**DOI:** 10.1155/2022/2565494

**Published:** 2022-07-02

**Authors:** Qiulong Zhao, Chunxue Wang, Jiaxin Cheng, Hui Yan, Ling Wang, Dawei Qian, Jinao Duan

**Affiliations:** ^1^Jiangsu Key Laboratory for High Technology Research of TCM Formulae, Nanjing 210023, China; ^2^Jiangsu Collaborative Innovation Center of Chinese Medicinal Resource Industrialization, National and Local Collaborative Engineering Center of Chinese Medicinal Resources Industrialization, Formulae Innovative Medicine, Nanjing University of Chinese Medicine, Nanjing 210023, China

## Abstract

According to the sixth edition of China's “New Coronavirus Diagnosis and Treatment Plan (NCDTP),” ReDuNing injection (RDN) was firstly introduced to treat severe and critical COVID-19, whereas its combination with broad-spectrum antibiotics was suggested to take with extreme caution and full reasons. Therefore, we aim to describe the pharmacokinetics of seven active phytochemicals and semiquantification of nine relevant metabolites in ReDuNing injection (RDN) after combining with cefuroxime sodium (CNa) for injection in rat plasma. Male Sprague–Dawley rats were randomly assigned to six groups, and they were intravenously administered, respectively, with different prescriptions of RDN (2 mL/kg) and CNa (225 mg/kg). At different time points (0.03, 0.08, 0.17, 0.24, 0.33, 0.50, 0.67, 1, and 6 h) after administration, the drug concentrations of iridoids glycosides, organic acids, and metabolites in rat plasma were determined using ultrahigh-pressure liquid chromatography coupled with linear ion rap-orbitrap tandem mass spectrometry (UHPLC–LTQ–Orbitrap–MS), and main pharmacokinetic parameters were estimated by noncompartment model. The results showed that there were differences in pharmacokinetic parameters, AUC_(0-t)_, T_1/2_, *C*_max_, CL of iridoids glycosides, and organic acids, after the intravenous administration of the different combinations of RDN and CNa. Moreover, different combinations of the injections also resulted in different curves of relative changes of each metabolite. The obtained results suggested that RDN and CNa existed pharmacokinetic drug–herb interactions in rats. The findings not only lay the foundation for evaluating the safety of RDN injection combined with CNa but also make contributions to clinically applying RDN injection combined with CNa, which works potentially against severe forms of COVID-19.

## 1. Introduction

Since December 2019, the outbreak of COVID-19 has caused over 196,000,000 confirmed cases with more than 4,100,000 deaths worldwide as of July 29, 2021, according to statistical data from the WHO and Hopkins University website (https://nxw.so/5JfTZ). To fight against its ravages, more than 280 candidate vaccines are currently in development, 23 of which are already in phase 3 trials through different platforms [1]. However, COVID-19 also evolved into an “improved” version, i.e., the Delta variant identified in October 2020 in India [2], with ∼60% more transmissible properties than the already highly infectious Alpha variant [3]. As a result, a single dose of either Pfizer or AstraZeneca vaccines barely induced neutralizing antibodies against the Delta variant in individuals who were not previously infected with SARS-CoV-2 [4]. Some scholars think that it is merely a matter of time before the Delta becomes dominant and takes over, however, the hope is to slow its expansion through vaccination [3]. In addition, severe systemic events (vomiting, diarrhea, fatigue, headache, chills, muscle pain, joint pain, etc.) were reported when using the above two vaccine candidates [5]. Although a second dose of the AstraZeneca vaccine boosted protection against Delta to 60% [3], it remains worrisome and uncertain what the vaccine efficacy against Delta will be for those more severe forms of disease [6].

If the above uncertainty occurs, what can we do for severe forms of the disease? According to the sixth edition of China's “New Coronavirus Diagnosis and Treatment Plan (NCDTP)” (https://nxw.so/5Oo6e), ReDuNing injection (RDN) was first recommended to treat severe and critical COVID-19. RDN, a patented traditional Chinese medicine formulation containing *Gardeniae jasminoides* E. (123), *Lonicera japonica* T. (261), and *Artemisia annua* L (151), aggregating 535 compounds (potentially repetitive, https://tcmsp-e.com/), has been widely used as an antipyretic and anti-inflammatory drug to treat the common cold, cough, acute upper respiratory infection, and acute bronchitis [7, 8]. Iridoid glycosides and organic acids are the two main phytochemicals of RDN [9–11]. Geniposide, shanzhiside, genipin-1-*β*-gentiobioside, secoxyloganin, neochlorogenic acid, chlorogenic acid, and cryptochlorogenic acid are important bioactive components [12–14]. Based on network pharmacology and molecular docking, Bei Yin found that the key ingredients from RDN have good binding power with SARS-CoV-2 3CL hydrolase and ACE_2_ by acting on oxidative stress response pathways [15], the MAPK signaling pathway, and the chemokine pathway.

Currently, several clinical reports have involved RDN against bronchitis and pneumonia in combination with cefuroxime sodium for injection (CNa, a semisynthetic cephalosporin with relatively broad-spectrum antimicrobial activity) [16–19], which effectively inhibits viruses or bacteria, removes inflammatory mediators, regulates patient immunity, and improves patient symptoms [20, 21]. However, such a combination may result in severe medicinal accidents because of the *in vivo* drug–herb interaction causing changes in the blood concentration and metabolites [22–26], to which we should pay attention in the medication guide of RDN (https://nxw.so/5x4Pz).

To ensure safety and efficacy, qualitative and quantitative analyses of RDN-derived major components have been processed using near-infrared spectroscopy and UPLC methods [27, 28]. In addition, there have been several reports on *in vivo* pharmacokinetic studies of some iridoid glycosides and organic acids, such as geniposide, neochlorogenic acid, chlorogenic acid, and cryptochlorogenic acid, in RDN-administered humans or rats [29–34]. Although most research has made efforts to develop an effective method to study the *in vivo* profile of prototypes in RDN, knowledge on not only the pharmacokinetics of RDN combined with other drugs but also the change trends of relevant metabolites are still limited. Given the absence of *in vivo* studies reporting RDN injection combined with CNa, we aimed to evaluate the changes in the pharmacokinetic profiles of prototypes and major metabolites of RDN with CNa after coadministration. This study mainly focused on the interaction of the two injections with regards to pharmacokinetics using UHPLC-LTQ-Orbitrap-MS, laying the foundation of drug combination of RDN injection with CNa and making a contribution to preparing a potential therapeutic for severe forms of COVID-19.

## 2. Materials and Methods

### 2.1. Chemicals and Reagents

**Figure 1:**
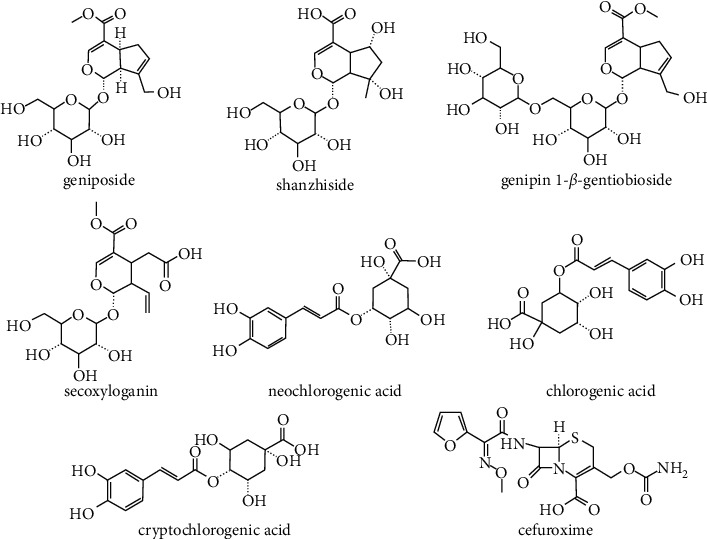
The chemical structures of seven ingredients of RDN and cefuroxime.

**Figure 2:**
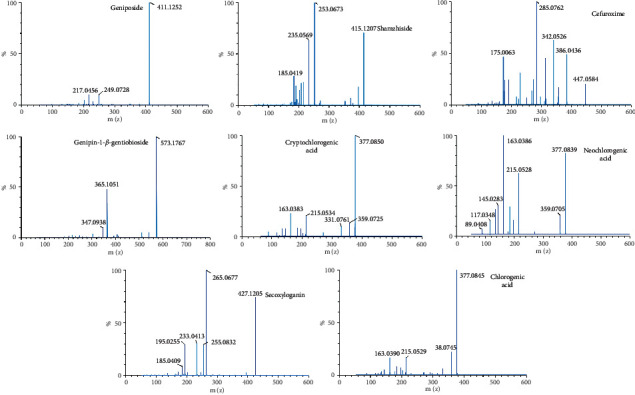
The mass spectrum of seven ingredients of RDN and cefuroxime.

RDN injection was provided by the Jiangsu Kanion Pharmaceutical Co., Ltd, and CNa was obtained from the Shenzhen Lijian Pharmaceutical Co., Ltd. Reference standards (geniposide, shanzhiside, genipin-1-*β*-gentiobioside, secoxyloganin, neochlorogenic acid, chlorogenic acid, and cryptochlorogenic acid) were provided by the Shanghai Rongqi Pharmaceutical Technology Co., Ltd. (Shanghai, China). Clarithromycin (IS, 98% purity, 1529013) was purchased from the Shanghai Aladdin Bio-Chem Technology Co., Ltd. Cefuroxime was obtained from the National Institutes for Food and Drug Control. The relevant chemical structures are shown in Figure 1. The mass spectrum of seven ingredients of RDN and cefuroxime are shown in Figure 2. Acetonitrile, methanol, and formic acid were of HPLC grade from Merck (Darmstadt, Germany). Deionized water was purified by a Milli-Q system (Millipore, Billerica, MA, USA). The other chemical reagents were of analytical grade.

A total of 7 components in RDN injection were determined. The mean content of each phytochemical was as follows: 9.95 mg/mL of geniposide, 0.287 mg/mL of shanzhiside, 5.34 mg/mL of genipin-1-*β*-gentiobioside, 1.13 mg/mL of secoxyloganin, 3.96 mg/mL of neochlorogenic acid, 6.33 mg/mL of chlorogenic acid, and 4.07 mg/mL of cryptochlorogenic acid.

### 2.2. Instrument and Chromatographic Conditions

An LTQ-Orbitrap Velos mass spectrometer (Thermo Scientific, Hemel Hempstead, UK) equipped with an ESI source was applied to acquire profile mass spectra. Liquid chromatographic separations were carried out using a UHPLC Dionex Ultimate 3000 (Thermo Scientific, San Jose, USA) and an ACQUITY UPLC BEH T3 column (1.7 *μ*m, 2.1 mm × 100 mm). The mobile phase consisted of water/0.1% formic acid (solvent A) and acetonitrile (solvent B) at a constant flow rate of 0.4 mL/min, and the injection volume was 2 *μ*L. Separation was carried out within 21 min under the following conditions: 0–3 min, 1% B; 3–9 min, 1 ⟶ 10% B; 9–12 min, 10 ⟶ 20% B; 12–13 min, 20 ⟶ 25% B; 13–15 min, 25% B; 15–18 min, 25 ⟶ 40% B; 18–20 min, 40 ⟶ 99% B. The column was equilibrated for 1 min prior to each analysis, and the related MS data of seven prototypes and cefuroxime are shown in Table 1.

The optimized ESI operating parameters were as follows: source voltage, 5 kV; sheath gas, 40 (arbitrary units); auxiliary gas, 15 (arbitrary units); heater temperature and capillary temperature, 350°C.

### 2.3. Preparation of Stock and Standard Solutions

To prepare the stock solutions [35], about 1 mg (geniposide, shanzhiside, genipin-1-*β*-gentiobioside, secoxyloganin, neochlorogenic acid, chlorogenic acid, and cryptochlorogenic acid), as well as the IS, and 2 mg (cefuroxime) were individually dissolved in methanol-water (50 : 50, v/v). The final concentration of cefuroxime stock solution was 2.0 mg/mL, while the other seven prototypes with IS were at 1.0 mg/mL [36]. The stock solutions of all analytes were combined and further diluted with methanol water (50 : 50, v/v) using 2, 5, 2, 5, 2 and 5-fold serial gradient. 200 *μ*L of the combined working solution was added to 50 *μ*L plasma and 20 *μ*L IS (1 *μ*g·mL^−1^) to obtain the calibration standards at 62, 310, 619, 3095, 6190, 30,952, and 61,904 ng/mL for geniposide, 8, 39, 78, 389, 779, 3893, and 7786 ng/mL for shanzhiside, 25, 125, 250, 1248, 2495, 12,476, and 24,952 ng/mL for genipin-1-*β*-gentiobioside, 26, 129, 258, 1288, 2575, 12,876, and 25,752 ng/mL for secoxyloganin, 25, 126, 252, 1259, 2519, 12,595, and 25,190 ng/mL for neochlorogenic acid, 30, 150, 300, 1500, 3000, 15,000, and 30,000 ng/mL for chlorogenic acid, 22, 109, 218, 1088, 2175, 10,876, and 21,752 ng/mL for cryptochlorogenic acid, and 557, 2785, 5570, 27,850, 55,700, 278,500, and 557,000 for cefuroxime, respectively. QCs were separately prepared using the same way at three different concentration levels, including the low-quality control (77, 10, 31, 32, 31, 38, 27, and 696 ng/mL for the above eight analytes), middle-quality control (3095, 389, 1248, 1288, 1260, 1500, 1088, and 27,850 ng/mL for the above eight analytes), and high-quality control (49,523, 6229, 19,962, 20,602, 1260, 24,000, 17402, and 445,600 ng/mL for the above eight analytes).

### 2.4. Sample Preparation

A 50 *μ*L aliquot of plasma was mixed with 200 *μ*L of methanol and 20 *μ*L of IS (2) [37]. The solution was vortexed for 2 min and centrifuged at 13,000 rpm for 10 min. The clarified supernatant was transferred to a new polypropylene tube and evaporated to dryness under nitrogen at 30°C. The residue was reconstituted in 50 *μ*L acetonitrile-water (5 : 95 v/v), vortex-mixed, and centrifuged again under the above-mentioned conditions [38]. 2 *μ*L of this solution was injected into the UPLC-MS/MS for analysis [39].

### 2.5. Method Validation of Prototypes

The proposed quantitative method was validated by determining the selectivity, linearity, precision, accuracy, extraction recovery, matrix effect, and stability according to the guidance of the Food and Drug Administration (FDA) for the validation of bioanalytical methods [40].

#### 2.5.1. Selectivity

The selectivity of the method was evaluated by comparing the chromatograms of blank plasma samples with those of corresponding standard samples spiked with analytes, IS, and samples.

#### 2.5.2. Linearity and Lower Limit of Quantification

The linear calibration curves of seven analytes with cefuroxime were determined by plotting the peak area ratio (*y*) of analytes to IS versus the analyte concentration (*x*) by least-squares linear regression using 1/*x*^2^ as the weighing factor. The calibration curves had to have a correlation coefficient (*R*) of 0.99 or better. The limit of detection (LLOD) and quantification (LLOQ) were determined at signal-to-noise ratios of 3 and 10 by analyzing the standard plasma samples.

#### 2.5.3. Precision and Accuracy

The precision and accuracy depended on analyzing QC samples at three different concentration levels (low, medium, and high) in six replicates. The RSD was used for reporting precision. The accuracy was established by comparing the measured concentration with its true value. Accuracy and precision were assessed by the relative error (RE) and relative standard deviation (RSD), respectively. Precision should not exceed 15%, and the accuracy should be within ±15% for the QC samples.

#### 2.5.4. Extraction Recovery and Matrix Effect

The extraction recovery of the seven prototypes with cefuroxime at three QC levels was evaluated by comparing the peak area of each analyte extracted from plasma QC samples with those latter extracted from the blank matrix (*n* = 6). The matrix effect was determined as the peak area of the analytes dissolved in the blank matrix versus that dissolved with methanol solution [41].

#### 2.5.5. Stability

The stability of seven analytes with cefuroxime in plasma was evaluated by keeping the low-, medium-, and high-QC samples at 25°C for 4 h (short-term stability), storing the samples at −20°C for 21 days (long-term stability), and undergoing three freeze/thaw cycles. The autosampler stability was evaluated by analyzing QC samples at 4°C for 24 h.

### 2.6. Pharmacokinetic Study

#### 2.6.1. Animals

All Sprague–Dawley rats (male, weighing 220 ± 20 g, purchased from Nanjing Jiangning District Qing long shan animal breeding farms, Jiangsu Province, License No. SCXK-2017-0001) were specific pathogen-free. The rats were acclimated for at least a week at room temperature (24 ± 1°C) in a light-controlled environment (12/12 h light/dark cycle) with free access to standard chow and water. They underwent 12 h of fasting prior to the experiment. Animal welfare and experimental procedures were consistent with the Guide for the Care and Use of Laboratory Animals National Research Council (U.S.) committee for the Update of the Guide for the Care and Use of Laboratory Animals (2011) and related ethical regulations of the Nanjing University of Chinese Medicine.

#### 2.6.2. Animal Treatment

Experimental animals were randomly divided into eight groups (six rats per group), which are listed in Table 2. The doses of RDN and CNa based on clinical practice were 2 mL/kg and 225 mg/kg, respectively. Groups 1/2/3 received RDN/CNa/coadministration (RDN and CNa) alone merely for one day, while Groups 4/7/8 received RDN/CNa/coadministration (RDN and CNa) for 5 consecutive days. Group 5 received CNa on the first 4 days and then RDN on the 5^th^ day. Group 6 received 4-day coadministration of RDN and CNa and RDN alone on the last day. All of the above drugs were administered in the form of intravenous injection through the caudal vein, and there was no interval between drug-herb administration [42]. Serial blood samples (300 *μ*L) were obtained on the last day at 0 (predose), 0.03, 0.08, 0.17, 0.24, 0.33, 0.50, 0.67, 1, and 6 h after intravenous injection. After centrifugation at 4500 rpm for 10 min, plasma was collected and frozen at −20°C until analysis.

#### 2.6.3. Statistical Analysis

Pharmacokinetic parameters were determined using Drug and Statistic (DAS) software (version 2.0, Chinese Pharmacological Society). Parameters, including the terminal elimination half-life (T_1/2_), maximum plasma concentration (*C*_max_), area under the plasma concentration-time curve (AUC_0−t_), and plasma clearance (CL), were determined. Data were presented as the mean ± standard deviation (SD). Student's *t*-test was used for the comparisons of two groups, except when the variances of the compared groups were not homogeneous, in which case the Mann–Whitney *U* test was employed.

### 2.7. Semiquantificative Method Validation for RDN Metabolites

#### 2.7.1. Identification of Metabolites

The metabolites were identified by comparing the retention time, precise molecular mass, and MS/MS data of blank plasma and dosed plasma, which had been reported by Acta Pharmaceutical Sinica [43].

#### 2.7.2. Method Validation for the Semiquantification of Metabolites

*(1) Linear Range*. A semiquantitative method for nine metabolites was built because of the lack of reference standards. Samples from every time point were mixed together (150 *μ*L per sample) as the mother solution [44]. The mother solutions of 5 *μ*L, 10 *μ*L, 20 *μ*L, 40 *μ*L, 80 *μ*L, 120 *μ*L, and 200 *μ*L were added to 1.5 mL centrifuge tubes, and then, 200 *μ*L of each tube was added to 200 *μ*L of blank plasma. Every tube was supplemented with 80 *μ*L of IS solution (1 *μ*g/mL) and 400 *μ*L of methanol. Then, 600 *μ*L of supernatant was collected after centrifugation at 13,000 rpm for 10 min and concentrated by centrifugation. Next, the residue was dissolved by 50 *μ*L of 5% acetonitrile. These seven samples aimed to establish the linear range of each metabolite curve. Samples with 15 *μ*L, 40 *μ*L, and 160 *μ*L of mother solution were used as QC samples.

*(2) Precision and Stability*. The precision of metabolites was evaluated by measuring QC samples at low, medium, and high concentrations, expressed as the RSD, which should not exceed 15%. The stability of metabolites was estimated as described in “Section 2.5.5.”

### 2.8. Curves of Relative Changes for the Metabolites

The curves of relative changes of these metabolites during 6 h after intravenous injection were constructed by plotting the peak area ratios of metabolites to the IS (*X*-axis is time and *Y*-axis is the peak area ratios of metabolites to IS), respectively.

## 3. Results

### 3.1. Method Validation of Prototypes

#### 3.1.1. Selectivity

**Figure 3:**
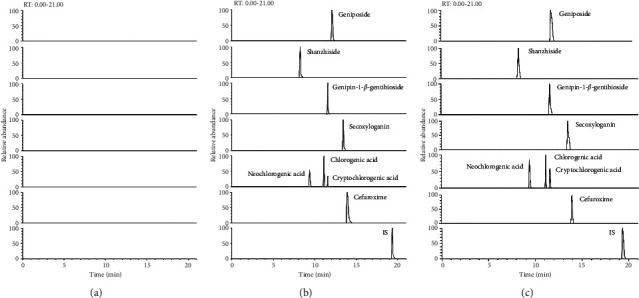
The representative ion chromatograms of seven analytes, cefuroxime, and clarithromycin (IS): (a) blank plasma, (b) blank plasma spiked with seven analytes, cefuroxime, and IS, and (c) medicated plasma sample.

The protein precipitation methodology through mass spectrometry detection presented good selectivity for the analytes. Typical chromatograms obtained from a blank, a spiked plasma sample with the seven analytes, cefuroxime, and the IS after an intravenous dose are shown in Figure 3. Significant interference from endogenous components was hardly found to affect the detection of the analytes and IS in all samples.

#### 3.1.2. Linearity and Lower Limit of Quantification

Regression equations, linear ranges, correlation coefficients and LLOQs for the seven analytes with cefuroxime are shown in Table 3. The assay exhibited good linearity for all constituents with correlation coefficients in the range from 0.9990 to 0.9996.

#### 3.1.3. Extraction Recoveries and Matrix Effects

The results of the matrix effect and extraction recovery are summarized in Table 4. The recoveries of the analytes extracted from the plasma at the three QC concentration levels were 90.36–97.73% for geniposide, 86.91–97.74% for shanzhiside, 88.60–95.27% for genipin-1-gentiobioside, 87.55–95.49% for secoxyloganin, 87.94–92.70% for neochlorogenic acid, 92.48–93.29% for chlorogenic acid, 88.47–92.56% for cryptochlorogenic acid, and 87.62–101.41% for cefuroxime. The recovery of the IS was 94.27%. The matrix effects of the analytes were in the range of 86.84–94.40% with RSD values below 10%, and the matrix effect of the IS was 96.47%. These results suggested that the effect of the matrix on the quantification of the contents of RDN was negligible.

#### 3.1.4. Precision and Accuracy

The precision and accuracy were confirmed by assaying the QC samples at three concentration levels, as listed in Table 4. Both the intraday and interday precision of the QC samples in the plasma were below 11.49% at each level, and the accuracy of chemical ingredients ranged from −7.73% to 9.22%. All of these values were within the acceptable range, and the method was judged to be suitably accurate and precise.

#### 3.1.5. Stabilities

The results of the stability study, as shown in Table 5, suggested that the analytes in the plasma maintained good short-term stability, long-term stability, freeze-thaw stability, and autosampler stability.

### 3.2. Pharmacokinetic Study of Prototype Compounds

**Figure 4:**
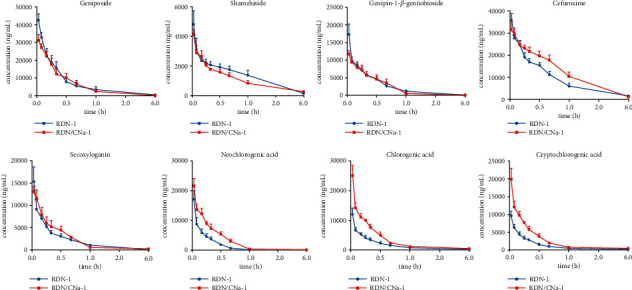
The mean plasma concentration-time profiles of seven analytes with cefuroxime after single coadministration of RDN and CNa.

**Figure 5:**
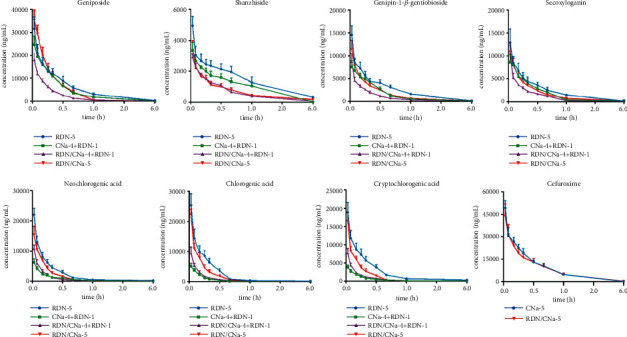
The mean plasma concentration-time profiles of seven analytes with cefuroxime after multiple coadministration of RDN and CNa.

The above validated UHPLC–LTQ–Orbitrap–MS method was applied successfully to the pharmacokinetic study in the rat plasma for the respective groups. The plasma concentration-time profiles of seven analytes with cefuroxime in single combination and multiple combination are illustrated in Figures 4 and 5, respectively. The pharmacokinetic parameters are shown in Table 6.

### 3.3. Method Validation for the Semiquantification of Metabolites

#### 3.3.1. Identification of Metabolites

**Figure 6:**
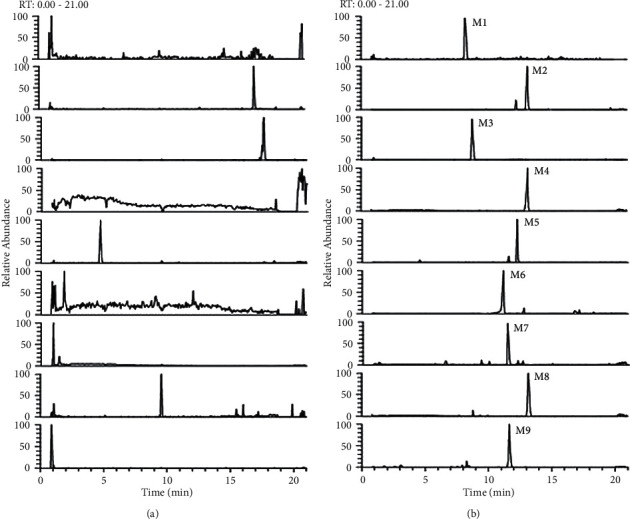
The representative extract ion chromatograms of nine metabolites: (a) blank plasma and (b) 1 h plasma sample after intravenous administration of RDN.

**Figure 7:**
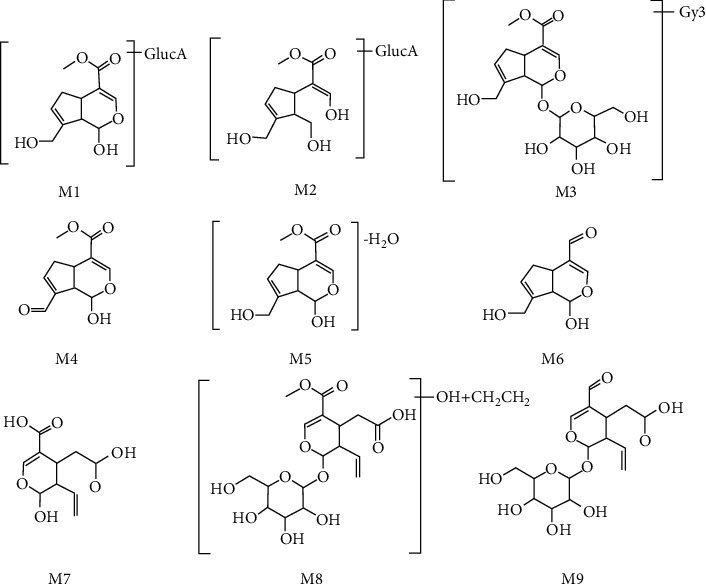
The structures of metabolites in rat plasma.

Nine metabolites of geniposide and secoxyloganin were identified with MS data. Representative extract ion chromatograms and MS data of metabolites are shown in Figure 6 and Table 7, respectively. The structures of metabolites are shown in Figure 7.

#### 3.3.2. Semiquantitative Method for Metabolites

The proposed semiquantitative method was validated by linear range, precision, and stability tests, and the results are shown in Tables 8 and 9. The method satisfied the demands of semiquantification for the metabolites.

### 3.4. Curves of Relative Changes for Metabolites

**Figure 8:**
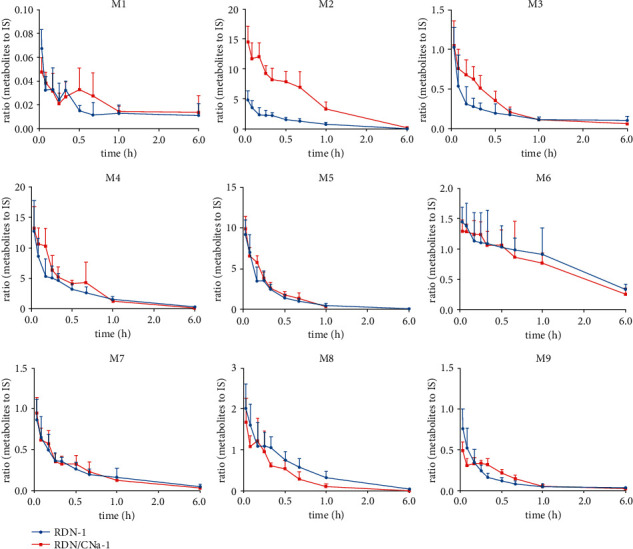
The curves of relative changes of the metabolites during 6 h after single coadministration.

**Figure 9:**
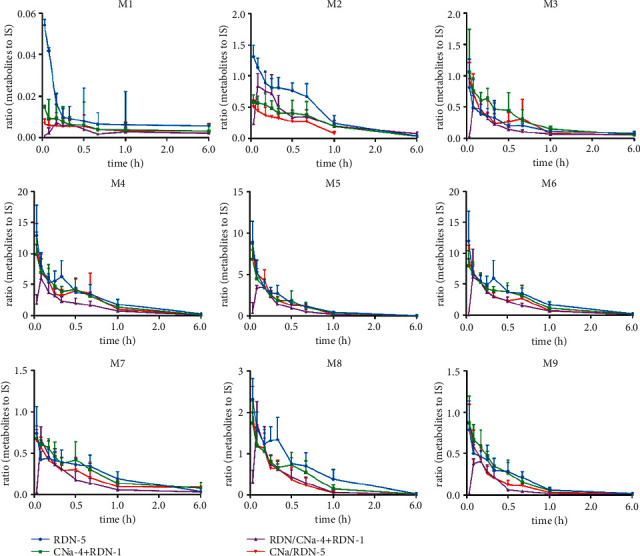
The curves of relative changes of the metabolites during 6 h after multiple combination.

The curves of relative changes of these metabolites during 6 h are displayed in Figures 8 and 9.

## 4. Discussion

### 4.1. LC–MS Optimization

To develop a sensitive method, all analytes were full-scanned by the positive and negative modes. It was found that the analytes could be ionized under both modes. The mass spectrometric parameters, such as the source voltage and heater temperature, capillary temperature, the flow rate of sheath gas, and the flow rate of auxiliary gas, were optimized to obtain the highest signal for the precursor ions and product ions mentioned above.

### 4.2. Pharmacokinetics of RDN by Comparing the Separate Administration of RDN with Single Coadministration of RDN and CNa (Group 1 and Group 3)

Iridoid glycosides in plasma showed a converse trend to organic acids after combination with CNa in pharmacokinetics. The one-time coadministration of RDN and CNa led to lower plasma concentrations of iridoid glycosides, with reduced AUC_(0-t)_ and *C*_max_ values, respectively, especially geniposide. Compared with the administration of RDN alone (Group 1), the AUC_(0-t)_ and *C*_max_ values for geniposide in Group 3 were reduced by 14.5% and 26.8%, respectively. T_1/2_ values were shortened from 0.50 to 0.27 h, and the three parameters were considered to be significant (*p* < 0.05) after the analysis of variance. The differences in two parameters, AUC_(0-t)_ and *C*_max_, of organic acids (neochlorogenic acid, chlorogenic acid, and cryptochlorogenic acid) in plasma were appreciably increased by 1.2–2.1 times after injecting RDN combined with CNa, and CL values also decreased significantly (*p* < 0.05). Meanwhile, the T_1/2_ was also decreased, however, there were no remarkable disparities in the values. CNa is not metabolized and is principally excreted unchanged in urine in terms of both glomerular filtration and tubular secretion [45, 46]. Such cephalosporin tubular reabsorption across brush-border membranes is mediated by pH-dependent peptide transporters PEPT1, PEPT2, and OAT [46–48]. As a good substrate of the above relevant transporters, CNa at higher concentrations can competitively block the uptake of organic acids with similar renal elimination [47–49], resulting in and adjunctively enhancing the blood level of organic acids [50].

### 4.3. Pharmacokinetics of RDN by Comparing the 5-Day Separate Administration of RDN with the 5-Day Coadministration of RDN and CNa (Group 4 and Group 8)

The plasma concentrations of iridoid glycosides and organic acids in rat serum were measured on the 5^th^ day and were lower in the presence of CNa (Group 8) than in its absence (Group 4). In the case of AUC_(0-t)_, the reductions of shanzhiside, genipin-1-*β*-gentiobioside, and secoxyloganin in rat plasma were up to 51.3% compared with the 5-day administration of RDN (*p* < 0.05), while geniposide had no significant changes. The trend of the AUC_(0-t)_ parameter in organic acids was opposite to that observed when the two drugs were coadministered once (Group 1 and Group 3). Compared with the 5-day RDN administration, the AUC_(0-t)_ values of the three chlorogenic acid isomers were reduced by nearly 30% under 5-day coadministration. Moreover, the *C*_max_ of neochlorogenic acid in Group 8 declined by 30.3%, which was more than two times higher than those of chlorogenic acid and cryptochlorogenic acid. Similar to the first part pharmacokinetics, there were also no significant changes in the T_1/2_ values of these isomers.

### 4.4. Pharmacokinetics of RDN by Comparing One Coadministration with 5-Day Coadministration (Group 3 and Group 8)

Compared with Group 3 (RDN/CNa-1), the concentration-time profiles of analytes in RDN showed faster elimination after intravenous administration in rats in Group 8 (RDN/CNa-5). The pharmacokinetic parameters of iridoid glycosides showed a 22.8–40.1% decrease in AUC_(0-t)_ and a 28.0–85.6% increase in CL as a consequence of combination with CNa. A 5-day coadministration of RDN and CNa decreased the AUC_(0-t)_, T_1/2_, and *C*_max_ of the three chlorogenic acid isomers, and the largest was up to 50.9%, while it significantly increased CL values by 72.8–100.6% (*p* < 0.05) compared with the single coadministration.

### 4.5. Pharmacokinetics of RDN by Comparing a 4-Day Administration of RDN and 1-Day CNa with a 5-Day Administration of RDN (Group 4 and Group 5)

In contrast to Group 4, the 5-day administration cycle of Group 5 consisted of 4-day RDN and 1-day CNa to fully simulate the combined administration in the clinic. The AUC_(0-t)_, *C*_max_, and CL of iridoid glycosides declined significantly (*p* < 0.05). The T_1/2_ values of dosed geniposide, shanzhiside, and genipin-1-*β*-gentiobioside were 0.19–0.23 h in Group 5, which were significantly lower than those of Group 4 (T_1/2_ > 0.36). The pharmacokinetic parameters, including AUC_(0-t)_ and *C*_max,_ in Group 5, displayed a 3-fold decrease for neochlorogenic acid, a 5-fold decrease for chlorogenic acid, and a 5-fold decrease for cryptochlorogenic acid. The rates of the decline were more than twice those of iridoid glycosides, indicating that CNa mostly affected the organic acids.

### 4.6. Pharmacokinetics of RDN by Comparing a 4-Day Coadministration and 1-Day RDN with 5-Day Separate Administration (Group 4 and Group 6)

The rats in Group 6 were treated with RDN combined with CNa on the first four days and administered RDN on the 5^th^ day. The AUC_(0-t)_ values of geniposide, shanzhiside, genipin-*β*-1-gentiobioside, and secoxyloganin in Group 6 decreased by nearly half those of Group 4. Except for secoxyloganin, the T_1/2_ and *C*_max_ of the other three iridoid glycosides significantly (*p* < 0.05) decreased. Similar to the iridoid glycosides, the pharmacokinetic parameters showed decreases of 62.0–70.8% in the AUC_(0-t)_ of organic acids and decreases of 50.7–62.8% in *C*_max_ compared with Group 4.

### 4.7. Influence of Coadministration with CNa on the Pharmacokinetic Profiles of Iridoid Glycosides, Organic Acids, and Metabolites in RDN

Drug combinations could significantly influence the blood concentrations and the pharmacokinetic parameters of the individual components after intravenous administration. In this study, the obtained pharmacokinetic parameter data for iridoid glycosides and organic acids in different groups showed differences.

The above results suggest that the systemic exposure (AUC_(0-t)_ and *C*_max_) of geniposide, shanzhiside, genipin-1-*β*-gentiobioside, and secoxyloganin were all weakened, and the elimination increased under the different coadministration conditions. CNa could be similar to its series cefetamet-inducing CYP3A4 enzyme activity [51], resulting in the fast metabolism of iridoid glycosides in rat plasma, however, further experiments are needed to prove this hypothesis. Meanwhile, CNa also affected the relative changes of the metabolites from geniposide and secoxyloganin. The ring-opening, dehydration, and hydroxymethylene loss metabolites achieved lower *C*_max_ values than others when coadministered with CNa one time. In Group 6 (RDN/CNa-4 + RDN-1), the peak time lag phenomenon was more obvious, which indicated that 4-day coadministration might inhibit these metabolic pathways *in vivo.*

The general trends of AUC_(0-t)_ and *C*_max_, two important parameters of neochlorogenic acid, chlorogenic acid, and cryptochlorogenic acid in rat plasma, increased after the intravenous administration of RDN combined with CNa, and CL values decreased compared with RDN alone, indicating that single coadministration might improve the bioavailability of organic acids. As reported, CNa is not metabolized and is excreted unchanged by the renal anionic transport system, principally in urine by both glomerular filtration and tubular secretion [45, 52]. Organic acids are negative ions and are mainly eliminated by the kidneys, which can cause competitive inhibition with cefuroxime from the blood to the kidney [53], which might be the reason for extending the exposure time after a single coadministration.

The results of the groups administered for 5 days inferred that long-term drug interactions could increase the elimination of organic acids in rat plasma, indicating that CNa might shorten the potency of organic acids *in vivo*. It was reported that combining antibiotics for a long time can cause bacterial group maladjustment *in vivo* [54, 55].

### 4.8. Influence of Coadministration with RDN on the Pharmacokinetic Profile of CNa

Compared with Group 2, the concentration-time profiles of CNa showed faster elimination after the intravenous administration of rats in Group 3 (RDN/CNa-1). The pharmacokinetic parameters of CNa showed an 11.4–33.0% decrease in AUC_(0-t)_ and a 9.9–72.5% increase in CL (all *p* < 0.05) as a consequence of a single combination with CNa. In addition, a 5-day coadministration with RDN (Group 8), also similar to the above single administration, decreased the AUC_(0-t)_ and increased the CL of CNa compared with Group 7 but with no significant difference. Some published papers have reported that genipin enhances Mrp-2-mediated bile formation and organic anion transport [56], through which approximately 45% of the antibiotic cefuroxime is eliminated renally [57], while iridoid glycoside and geniposide can promote bile secretion [58, 59], through which the cumulative percentage of cefuroxime reaches 12.83% [60].

## 5. Conclusion

A highly sensitive method using ultrahigh-pressure liquid chromatography coupled with linear ion trap-Orbitrap tandem mass spectrometry (UHPLC–LTQ–Orbitrap–MS) has been developed and validated to characterize the RDN and CNa drug–herb pharmacokinetics and semiquantification of RDN metabolites in rat plasma. The results demonstrated that RDN combined with CNa could lead to lower systemic exposure of iridoid glycosides, while one coadministration could lead to the slower elimination of organic acids but faster elimination in 5-day different combinations of coadministration. The curves of the relative changes of nine metabolites of geniposide and secoxyloganin over 6 h were obtained. The pharmacokinetic results not only lay the foundation for evaluating the safety of RDN injection combined with CNa but also contribute to clinically applying RDN injection combined with CNa, which potentially protects against severe forms of COVID-19.

## Figures and Tables

**Table 1:** The related MS data of seven prototypes with cefuroxime.

Analyte	*t*_*R*_ (min)	Ionization mode	(*m/z*)	(ms/ms)
Geniposide	12.25	ESI^+^	411.1252	249.0728, 217.0456
Shanzhiside	8.49	ESI^+^	415.1207	253.0673, 235.0569, 185.0419
Genipin-1-*β*-gentiobioside	11.58	ESI^+^	573.1767	365.1051, 347.0938
Secoxyloganin	13.14	ESI^+^	427.1205	265.0677, 233.0413, 195.0255
Neochlorogenic acid	9.20	ESI^+^	377.0839	359.0745, 215.0528
Chlorogenic acid	11.29	ESI^+^	377.0845	359.0745, 215.0529
Cryptochlorogenic acid	11.07	ESI^−^	377.0850	359.0725, 215.0534
Cefuroxime	13.84	ESI^+^	447.0584	386.0436, 342.0526, 285.0762
Clarithromycin	11.33	ESI^+^	748.4811	590.3869, 158.1172

**Table 2:** The experimental design of pharmacokinetic studies.

Group	1-D	2-D	3-D	4-D	5-D
1 (RDN-1)	—	—	—	—	a
2 (CNa-1)	—	—	—	—	b
3 (RDN/CNa-1)	—	—	—	—	a + b
4 (RDN-5)	a	a	a	a	a
5 (CNa-4 + RDN-1)	b	b	b	b	a
6 (RDN/CNa-4 + RDN-1)	a + b	a + b	a + b	a + b	a
7 (CNa-5)	b	b	b	b	b
8 (RDN/CNa-5)	a + b	a + b	a + b	a + b	a + b

^*∗*^ a = RDN 2 mL/kg b = CNa 225 mg/kg.

**Table 3:** The regression equations, linear ranges, LLODs, and LLOQs of the seven analytes and cefuroxime.

Analyte	Calibration curves	Correlation coefficient	Range (ng/mL)	LLOD (ng/mL)	LLOQ (ng/mL)
Geniposide	*y* = 0.0165*x*−0.0048	0.9996	62–61904	8.5	25.4
Shanzhiside	*y* = 0.0688*x* + 0.0031	0.9994	8–7786	0.8	2.3
Genipin-1-*β*-gentiobioside	*y* = 0.0163*x* + 0.0327	0.9995	25–24952	2.1	6.6
Secoxyloganin	*y* = 0.1029*x* + 0.0241	0.9993	26–25752	1.6	5.9
Neochlorogenic acid	*y* = 0.0551*x* + 0.0518	0.9994	25–25190	4.3	8.2
Chlorogenic acid	*y* = 0.0883*x* + 0.0418	0.9993	30–30000	4.6	13.3
Cryptochlorogenic acid	*y* = 0.1215*x* + 0.0386	0.9994	22–21752	2.7	7.4
Cefuroxime	*y* = 0.0504*x* + 0.0526	0.9990	557–557000	16.4	47.5

**Table 4:** The precision, accuracy, recovery, and matrix effect for seven analytes with cefuroxime in rat plasma (*n* = 6).

Analyte	Concentration (ng/mL)	Interday		Intraday		Recovery	Matrix effect
		RSD (%)	Accuracy RE (%)	RSD (%)	Accuracy RE (%)	(Mean ± SD, (%))	(Mean ± SD, (%))
Geniposide	77	3.80	2.52	7.16	−1.95	90.36 ± 3.64	92.79 ± 4.86
	3095	5.36	−2.82	4.60	−3.31	97.73 ± 4.72	93.31 ± 4.60
	49523	8.64	−1.26	7.56	5.53	94.78 ± 6.58	89.00 ± 2.12

Shanzhiside	10	10.68	9.10	7.39	−7.73	86.91 ± 2.57	90.62 ± 2.72
	389	9.32	−3.54	5.79	1.28	97.74 ± 4.89	87.28 ± 2.55
	6229	8.66	−1.09	3.54	2.23	89.09 ± 6.38	93.74 ± 3.55

Genipin-1-*β*-gentiobioside	31	11.49	2.59	4.41	−2.26	90.97 ± 5.80	92.20 ± 2.94
	1248	7.00	−3.90	4.02	2.67	88.60 ± 7.62	89.10 ± 4.99
	19962	5.25	3.77	7.49	−1.17	95.27 ± 4.80	94.40 ± 1.74

Secoxyloganin	32	9.40	2.44	6.44	4.28	92.15 ± 6.07	90.80 ± 3.89
	1288	5.50	−1.53	6.30	9.22	95.49 ± 7.38	86.84 ± 4.17
	20602	5.84	2.69	8.74	−3.11	87.55 ± 3.02	93.11 ± 3.45

Neochlorogenic acid	31	7.24	6.94	4.46	1.18	90.00 ± 6.09	89.72 ± 2.83
	1260	7.54	1.85	7.17	−1.86	87.94 ± 5.37	92.09 ± 3.72
	20152	5.53	−4.17	6.04	3.73	92.70 ± 6.38	93.07 ± 4.26

Chlorogenic acid	38	5.53	1.77	6.20	−1.52	92.53 ± 8.23	94.11 ± 3.10
	1500	6.11	1.93	7.19	2.80	92.48 ± 6.33	86.98 ± 4.64
	24000	8.23	−3.19	7.00	4.27	93.29 ± 7.44	91.74 ± 2.85

Cryptochlorogenic acid	27	8.95	5.95	6.20	−3.42	92.56 ± 5.29	91.42 ± 4.19
	1088	4.97	−1.57	8.36	1.91	91.36 ± 3.52	88.87 ± 3.97
	17402	7.18	1.48	7.44	−2.52	88.47 ± 5.30	89.28 ± 2.89

Cefuroxime	696	8.28	4.81	6.88	−4.24	101.41 ± 17.85	90.23 ± 2.98
	27850	6.22	−1.89	10.15	5.26	93.39 ± 3.98	89.51 ± 5.96
	445600	3.41	1.87	5.52	−1.21	87.62 ± 4.77	93.41 ± 1.84

**Table 5:** The stabilities of seven analytes with cefuroxime of QC samples in rat plasma under different storage conditions.

Analyte	Concentration (ng/mL)	Three freeze–thaw cycles (mean ± SD, (%))	Long-term stability −20°C for 21 d (mean ± SD, (%))	Short-term stability 25°C for 4 h (mean ± SD, (%))	Post-prepared sample 4°C for 24 h (mean ± SD, (%))
Geniposide	77	97.47 ± 4.39	97.81 ± 3.11	93.43 ± 3.99	97.78 ± 3.83
	3095	92.72 ± 4.92	89.66 ± 2.33	97.65 ± 3.28	95.15 ± 4.62
	49523	95.19 ± 5.96	91.13 ± 2.39	91.08 ± 3.69	100.12 ± 1.04

Shanzhiside	10	92.17 ± 5.06	96.72 ± 3.16	90.38 ± 2.82	92.34 ± 1.57
	389	94.14 ± 3.27	90.92 ± 2.53	93.13 ± 5.65	92.08 ± 4.82
	6229	100.24 ± 8.32	101.27 ± 10.32	95.45 ± 6.94	98.14 ± 5.68

Genipin-1-*β*-gentiobioside	31	92.76 ± 5.85	87.69 ± 1.34	92.05 ± 5.30	96.27 ± 4.76
	1248	93.24 ± 4.68	96.54 ± 3.95	90.51 ± 5.12	94.81 ± 7.35
	19962	100.83 ± 6.76	97.90 ± 7.67	98.10 ± 6.62	99.08 ± 9.76

Secoxyloganin	32	93.62 ± 3.57	92.98 ± 5.47	93.90 ± 4.76	92.39 ± 3.20
	1288	91.57 ± 4.35	94.47 ± 4.03	90.46 ± 4.78	96.68 ± 4.75
	20602	98.82 ± 4.92	101.32 ± 6.26	98.30 ± 6.69	95.82 ± 7.45

Neochlorogenic acid	31	94.30 ± 3.49	96.81 ± 4.27	93.31 ± 4.71	94.07 ± 3.66
	1260	95.90 ± 4.45	89.67 ± 2.12	92.58 ± 4.53	96.86 ± 3.03
	20152	88.15 ± 1.30	98.42 ± 8.36	88.22 ± 1.64	102.90 ± 5.17

Chlorogenic acid	38	92.50 ± 4.98	91.84 ± 4.02	94.70 ± 3.89	94.88 ± 4.33
	1500	93.12 ± 4.73	93.88 ± 7.12	90.85 ± 3.64	91.36 ± 5.08
	24000	87.96 ± 1.45	88.84 ± 2.13	90.49 ± 1.56	89.06 ± 1.73

Cryptochlorogenic acid	27	92.77 ± 4.18	93.64 ± 4.54	92.07 ± 3.53	93.42 ± 2.51
	1088	94.93 ± 3.86	94.95 ± 6.15	95.98 ± 4.68	95.95 ± 4.95
	17402	88.24 ± 1.79	90.04 ± 2.29	90.61 ± 1.16	90.32 ± 1.08

Cefuroxime	696.25	97.92 ± 3.89	93.58 ± 3.69	93.68 ± 4.78	96.73 ± 3.95
	27850	95.09 ± 5.07	97.51 ± 3.49	93.01 ± 6.87	89.93 ± 2.56
	445600	100.78 ± 8.87	101.16 ± 9.03	89.62 ± 1.44	100.74 ± 13.72

**Table 6:** The pharmacokinetic parameters of the compounds in rat plasma after intravenous coadministration of RDN and CNa.

Compound	Group	AUC_(0-t)_	T_1/2_	*C*_max_	CL
		(ng/mL *∗* h)	(h)	(ng/mL)	(L/h/kg)
Geniposide	1 (RDN-1)	15882 ± 2455	0.50 ± 0.13	42673 ± 3368	1266 ± 181
	3 (RDN/CNa-1)	13576 ± 568^a^	0.27 ± 0.03^a^	31243 ± 3242^a^	1461 ± 64^a^
	4 (RDN-5)	10735 ± 1306	0.36 ± 0.03	31584 ± 5073	1639 ± 215
	5 (CNA-4 + RDN-1)	8870 ± 541^d^	0.23 ± 0.05^d^	24702 ± 3662^d^	2129 ± 204^d^
	6 (RDN/CNA-4 + RDN-1)	4590 ± 604^e^	0.21 ± 0.02^e^	18042 ± 2915^e^	4268 ± 580^e^
	8 (RDN/CNA-5)	10482 ± 1137^c^	0.17 ± 0.07^b,c^	35357 ± 4441	1871 ± 237^c^

Shanzhiside	1 (RDN-1)	2926 ± 423	0.40 ± 0.04	4816 ± 908	193 ± 28
	3 (RDN/CNA-1)	2318 ± 216^a^	0.58 ± 0.08^a^	4124 ± 324	218 ± 43
	4 (RDN-5)	3089 ± 463	0.53 ± 0.10	4972 ± 578	175 ± 27
	5 (CNA-4 + RDN-1)	2251 ± 238^d^	0.26 ± 0.03^d^	3344 ± 541^d^	231 ± 47^d^
	6 (RDN/CNA-4 + RDN-1)	1392 ± 132^e^	0.38 ± 0.04^e^	2920 ± 202^e^	405 ± 40^e^
	8 (RDN/CNA-5)	1503 ± 104^b,c^	0.63 ± 0.09	3358 ± 643^b,c^	356 ± 23^b,c^

Genipin-1-*β*-gentiobioside	1 (RDN-1)	5454 ± 515	0.29 ± 0.07	17337 ± 2898	1802 ± 219
	3(RDN/CNA-1)	5025 ± 648	0.17 ± 0.03^a^	11673 ± 964^a^	2092 ± 261
	4 (RDN-5)	4878 ± 360	0.17 ± 0.03	14540 ± 1994	1757 ± 333
	5 (CNA-4 + RDN-1)	2844 ± 340^d^	0.41 ± 0.17^d^	7593 ± 1412^d^	3084 ± 651^d^
	6 (RDN/CNA-4 + RDN-1)	1894 ± 270^e^	0.23 ± 0.04^e^	8800 ± 1676^e^	5102 ± 873^e^
	8 (RDN/CNA-5)	3010 ± 245^b,c^	0.24 ± 0.03^b,c^	11448 ± 1954^b^	2857 ± 786^b,c^

Secoxyloganin	1 (RDN-1)	4363 ± 481	0.34 ± 0.07	15334 ± 3324	466 ± 49
	3 (RDN/CNA-1)	4319 ± 647	0.50 ± 0.14^a^	13063 ± 1283	347 ± 72^a^
	4 (RDN-5)	4414 ± 454	0.40 ± 0.08	12893 ± 2938	431 ± 33
	5 (CNA-4 + RDN-1)	3056 ± 369^d^	0.29 ± 0.10	8625 ± 1680^d^	546 ± 110^d^
	6 (RDN/CNA-4 + RDN-1)	2291 ± 293^e^	0.35 ± 0.04	10509 ± 1878	785 ± 82^e^
	8 (RDN/CNA-5)	2982 ± 337^b,c^	0.27 ± 0.06^b,c^	10878 ± 2123	645 ± 98^b,c^

Neochlorogenic acid	1 (RDN-1)	3432 ± 541	0.16 ± 0.03	17113 ± 2866	2223 ± 293
	3 (RDN/CNA-1)	6631 ± 443^a^	0.15 ± 0.06	21577 ± 2564^a^	1172 ± 89^a^
	4 (RDN-5)	4639 ± 413	0.23 ± 0.10	22059 ± 2157	1528 ± 205
	5(CNA-4 + RDN-1)	1401 ± 191^d^	0.25 ± 0.05	6228 ± 1209^d^	4958 ± 626
	6 (RDN/CNA-4 + RDN-1)	1762 ± 240^e^	0.13 ± 0.02^e^	10871 ± 1093^e^	4310 ± 505.1^e^
	8 (RDN/CNA-5)	3259 ± 301^b,c^	0.15 ± 0.02	15385 ± 2881^b,c^	2350 ± 202^b,c^

Chlorogenic acid	1 (RDN-1)	2973 ± 307	0.35 ± 0.20	12047 ± 2071	3378 ± 562
	3 (RDN/CNA-1)	6350 ± 420^a^	0.22 ± 0.10	25083 ± 3526^a^	1758 ± 240^a^
	4 (RDN-5)	5614 ± 644	0.14 ± 0.03	25312 ± 3764	2190 ± 294
	5 (CNA-4 + RDN-1)	1018 ± 134^d^	0.13 ± 0.02	5077 ± 965^d^	12262 ± 1517^d^
	6 (RDN/CNA-4 + RDN-1)	1639 ± 263^e^	0.17 ± 0.04	9417 ± 1926^e^	7507 ± 1183^e^
	8 (RDN/CNA-5)	3998 ± 341^b,c^	0.16 ± 0.06	22140 ± 1861	3038 ± 327^b,c^

Cryptochlorogenic acid	1 (RDN-1)	2358 ± 249	0.24 ± 0.04	9650 ± 1207	3039 ± 325
	3 (RDN/CNA-1)	5080 ± 265^a^	0.21 ± 0.06	19946 ± 3024^a^	1426 ± 142^a^
	4 (RDN-5)	4824 ± 577	0.19 ± 0.03	18894 ± 2701	1554 ± 185
	5 (CNA-4 + RDN-1)	894 ± 97^d^	0.21 ± 0.06	3856 ± 565^d^	8353 ± 1052^d^
	6 (RDN/CNA-4 + RDN-1)	1424 ± 192^e^	0.19 ± 0.01	7679 ± 1253^e^	5421 ± 768^e^
	8 (RDN/CNA-5)	3100 ± 370^b,c^	0.15 ± 0.06	16484 ± 2279^c^	2551 ± 396^b,c^

Cefuroxime	2 (CNa-1)	258567 ± 16978	0.36 ± 0.05	318320 ± 34982	780 ± 111
	3 (RDN/CNa-1)	199337 ± 14691^f^	0.41 ± 0.05	358284 ± 31456	1095 ± 94^f^
	7 (CNa-5)	197432 ± 16765	0.27 ± 0.09	492963 ± 47916	1136 ± 93
	8 (RDN/CNa-5)	184016 ± 17314	0.25 ± 0.04	438024 ± 81058	1226 ± 119

^a^ *p* < 0.05, Group 1 vs Group 3; ^b^*p* < 0.05, Group 3 vs Group 6; ^c^*p* < 0.05, Group 2 vs Group 6; ^d^*p* < 0.05, Group 3 vs Group 4; ^e^*p* < 0.05; Group 3 vs Group 5 and ^f^*p* < 0.05; Group 2 vs Group 7.

**Table 7:** The related MS data of nine metabolites.

Analyte	t_*R*_ (min)	*m/z*	(ms/ms)	Parent	Identification
M1	8.22	425.1075	249.0728, 231.0625, 199.0217	Geniposide	Glucuronidation
M2	13.16	427.1207	233.0789	Geniposide	Ring-opened in geniposide
M3	8.77	492.1520	330.1004, 312.0900	Geniposide	Cys conjugation
M4	13.14	225.0752	207.0651, 195.0594, 151.0755	Geniposide	Oxidation of geniposide aglycone
M5	12.23	209.0804	191.0705, 177.0543	Geniposide	Dehydration of geniposide aglycone
M6	11.04	197.0802	179.0703, 169.0858, 151.0753	Geniposide	Hydroxymethylene loss of geniposide aglycone
M7	11.42	229.0699	211.0601, 201.0748	Secoxyloganin	Hydrolysis of secoxyloganin aglycone
M8	13.12	451.1823	243.0860, 225.1123	Secoxyloganin	Hydroxylation and ethylation
M9	11.63	375.1275	357.1138, 347.1331, 195.0652	Secoxyloganin	Hydroxymethylene loss

**Table 8:** The regression equations and linear range of the metabolites.

Analyte	Regression equations	Correlation coefficient	Linear range
M1	*y* = 0.0005*x* + 0.0038	0.9982	0.0005–0.0562
M2	*y* = 0.0189*x* + 0.2929	0.9947	0.0578–15.1105
M3	*y* = 0.0012*x* + 0.0047	0.9984	0.0065–0.9873
M4	*y* = 0.0257*x* + 0.7887	0.9968	0.0799–23.2977
M5	*y* = 0.0324*x* + 0.0042	0.9976	0.1080–11.7411
M6	*y* = 0.0181*x* + 0.0069	0.9923	0.0671–14.1415
M7	*y* = 0.0023*x* + 0.1084	0.9928	0.0135–2.2764
M8	*y* = 0.0135*x* − 0.0773	0.9945	0.0395–10.8626
M9	*y* = 0.0016*x* + 0.0302	0.9963	0.0130–1.4125

**Table 9:** The precision and stability of the metabolites.

Analyte	Volume (*μ*L)	Interprecision (RSD (%))	Intraprecision (RSD (%))	Three freeze–thaw cycles	Auto sampler stability 4°C for 24 h	Short-term stability room stability 25°C for 4 h	Long-term stability −20°C for 21 d
M1	15	7.65	11.84	−6.33	8.70	−8.85	6.60
	40	5.75	5.34	4.98	−13.07	−10.72	2.74
	160	7.72	10.31	−5.20	10.95	8.17	−8.03

M2	15	12.46	3.82	−3.49	−5.60	−5.82	−3.63
	40	8.24	6.77	2.06	4.29	3.08	−2.61
	160	5.39	4.30	−3.42	−4.50	5.61	6.95

M3	15	11.75	5.38	1.70	−5.06	8.13	−7.61
	40	5.00	1.30	5.99	4.61	−1.21	1.99
	160	4.05	11.86	−8.23	7.42	8.57	2.20

M4	15	2.99	7.14	−7.87	8.00	−8.87	−5.08
	40	7.18	6.62	7.20	−6.57	4.41	8.74
	160	10.23	3.07	11.16	9.31	−7.50	−8.72

M5	15	3.12	2.64	7.88	−8.88	3.45	−6.52
	40	5.14	6.25	−9.03	−9.90	−5.83	−5.07
	160	2.31	5.74	7.71	5.18	7.60	7.66

M6	15	11.05	10.25	4.98	−7.22	−9.92	3.86
	40	4.34	7.52	−6.57	7.32	−5.31	−9.45
	160	6.74	6.63	−3.84	6.95	6.44	6.88

M7	15	10.06	3.54	2.16	−5.39	4.90	−9.58
	40	7.59	2.50	6.89	4.42	−3.49	−6.53
	160	3.38	9.02	−2.17	6.30	7.56	5.95

M8	15	6.78	6.61	4.74	−3.74	4.39	−4.31
	40	10.56	9.69	5.64	−2.55	−7.54	5.32
	160	2.02	4.53	−10.05	4.43	8.94	−2.85

M9	15	4.23	7.10	−5.97	8.16	7.46	7.87
	40	7.83	3.14	3.81	−3.20	−4.62	−5.92
	160	6.38	5.25	2.09	5.04	−5.60	4.98

## Data Availability

A majority of the data used in this research are included in the article. Other data can be made available upon request from the first author and corresponding author.
